# Birth of an oceanic spreading center at a magma-poor rift system

**DOI:** 10.1038/s41598-017-15522-2

**Published:** 2017-11-08

**Authors:** Morgane Gillard, Daniel Sauter, Julie Tugend, Simon Tomasi, Marie-Eva Epin, Gianreto Manatschal

**Affiliations:** Institut de Physique du Globe de Strasbourg, UMR7516, Université de Strasbourg/EOST, CNRS, 1 rue Blessig, Strasbourg, Cedex F-67084 France

## Abstract

Oceanic crust is continuously created at mid-oceanic ridges and seafloor spreading represents one of the main processes of plate tectonics. However, if oceanic crust architecture, composition and formation at present-day oceanic ridges are largely described, the processes governing the birth of a spreading center remain enigmatic. Understanding the transition between inherited continental and new oceanic domains is a prerequisite to constrain one of the last major unsolved problems of plate tectonics, namely the formation of a stable divergent plate boundary. In this paper, we present newly released high-resolution seismic reflection profiles that image the complete transition from unambiguous continental to oceanic crusts in the Gulf of Guinea. Based on these high-resolution seismic sections we show that onset of oceanic seafloor spreading is associated with the formation of a hybrid crust in which thinned continental crust and/or exhumed mantle is sandwiched between magmatic intrusive and extrusive bodies. This crust results from a polyphase evolution showing a gradual transition from tectonic-driven to magmatic-driven processes. The results presented in this paper provide a characterization of the domain in which lithospheric breakup occurs and enable to define the processes controlling formation of a new plate boundary.

## Introduction

**Figure 1:**
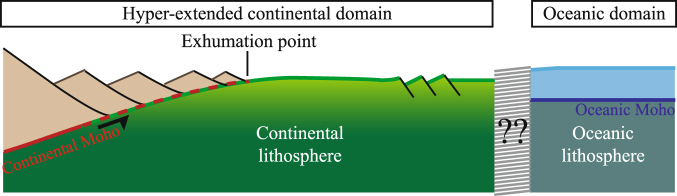
Cartoon highlighting the question about the architecture of the transition between continent-derived domains and unambiguous oceanic crust. Note that while the two domains are well described and understood, their transition is in most cases ill constrained and the processes that trigger breakup not constrained.

At rifted margins, a complex, heterogeneous continental crust faces a new, commonly layered, 6 to 7 km thick magmatic oceanic crust, entirely formed at a spreading center. While the range of compositions and the formation processes of these two fundamentally different types of crust are well documented, the transition from one to the other remains poorly understood. Some of the processes involved in this transition, such as exhumation of sub-continental mantle, are evidenced thanks to drilling^1,2^, field analogs^3,4^ and numerical modeling^5,6^. Recent studies also show that exhumed continental mantle can be affected by magmatic processes and/or subsequent normal and detachment faulting^7,8^, highlighting the complex polyphase tectono-magmatic evolution of this transitional domain. However, processes involved in the final transition between pre-existing continent-derived crustal and mantle rocks and newly accreted unambiguous oceanic crust remain enigmatic (Fig. 1). A main reason for this is the lack of good quality seismic data imaging the transition zone into unequivocal oceanic crust.

Classically, the first oceanic crust is mapped as a sharp boundary referred to as Continent-Ocean Boundary (COB) using different criteria such as the location of the oldest magnetic anomaly^9^, identification of Mid Oceanic Ridge (MOR) type magmatic rocks^10^, step ups or downs of top basement (e.g. outer high^11^), landward limit of typical oceanic Moho^12^, transition from wedge-type stratigraphic patterns to passive infill and the occurrence of a so called “Breakup Unconformity”^13^. However, these markers usually do not point the edge of first oceanic crust at the same location, revealing the ambiguity of COB definitions and its strong dependency on type and quality of used datasets^14,15^. Moreover, it remains debated^15^ if breakup is a fast, catastrophic event resulting in a sharp boundary, or, alternatively, if it is a gradual process resulting in a transitional domain, i.e. a diffuse plate boundary. For magma starved margins, it has been proposed that the process is rather transitional, leading to an embryonic^10,16^, proto-oceanic^7^, or outer domain^15^ that is generally associated with an area where magmatism increases significantly. However, up to now, no dataset clearly shows the architecture of this transition. Ref.^17^ suggested, based on magnetic modelling that the onset of oceanic spreading at the Newfoundland margin was linked to a major, post-exhumation magmatic event. However, there, seismic data cannot constrain this hypothesis and magnetic modelling results are non-unique^18^. On the contrary, ref.^8^ proposed, based on seismic observations, a gradual transition from tectonic to magma controlled processes at the Australian-Antarctic rifted margins.

In this study, we show two 2D seismic sections acquired by PGS (Petroleum Geo-Services) in the Gulf of Guinea. We consider them as the so far best images of this transitional domain. Based on these sections, we show the structural relationships between different types of basement, fault structures, sediments and magmatic processes that interact during lithospheric breakup and formation of first oceanic crust.

## Continental and oceanic domains

**Figure 2:**
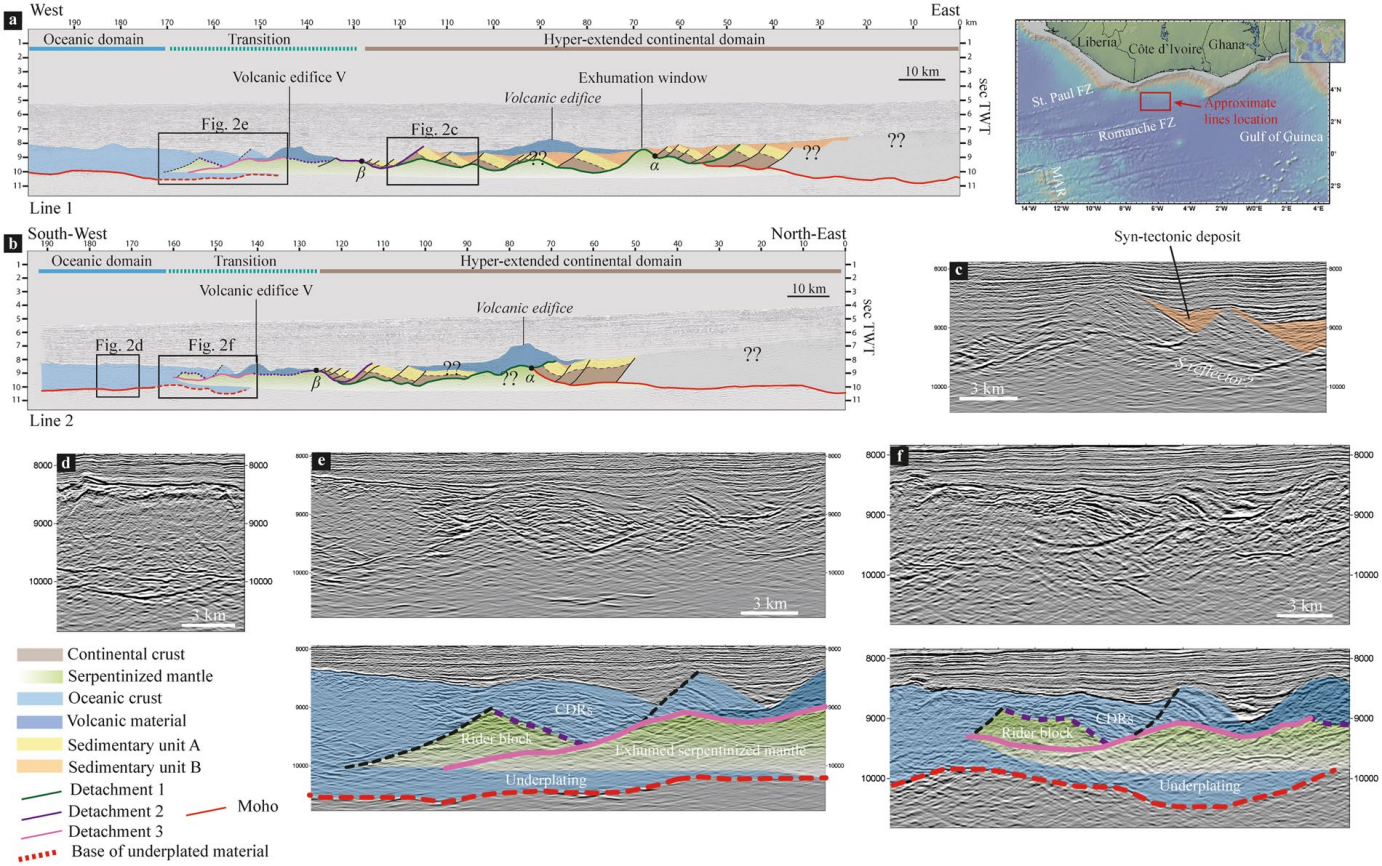
Interpreted seismic sections and location of the study area. (**a**) Interpreted Line 1. (**b**) Interpreted Line 2. The two seismic sections cross cut in the transitional domain. They display the location of the zooms, the main structural domains and points α (exhumation point) and β (end of continental block). (**c**) Zoom on the allochthon blocks of continental crust well displaying the “S reflector” and syn-tectonic deposits. (**d**) Zoom over the steady state oceanic crust. (**e**) and (**f**) show zooms of the transitional domain and associated interpretations particularly showing the Continentward Dipping Reflectors (CDRs) and magmatic underplating. The map has been created in using GeoMapApp 3.6.4 application. It is freely available on http://www.geomapapp.org/.

The Gulf of Guinea results from the separation between the African and South American plates. The structural evolution of this margin is driven by the development of several transform faults that initiated in Early Cretaceous time^19^. Following the separation of the continental crust, transform tectonics lead to the formation of divergent basins or pull-apart grabens, resulting in a highly segmented margin in late Albian time^20^. The two PSTM (Pre-Stack Time Migration) seismic sections displayed in this paper are located off the Ivory Coast coast, in between Saint Paul and Romanche fracture zones (Fig. 2). Line 1 is oblique relative to the opening direction while Line 2 is parallel.

The two seismic sections image the architecture of the margin from a hyper-extended continental domain to an undisputable oceanic crust (Fig. 2a,b). At the eastern part of the sections, strong reflections marking the base of the continental crust (continental Moho) are clearly visible at around 10 sec TWT. Several normal faults affect the crust and the overlying sediments (unit A) and root into Moho. The continental crust thins drastically toward the ocean and the Moho reflections reach the top basement, marking the exhumation point^21,22^ (Point α on Fig. 2a,b). However, several major tilted blocks are present oceanward and display pre-tectonic sediments similar to the unit A (Fig. 2c). These blocks are tilted above a major reflector (Fig. 2c) comparable to the “S reflection” observed at the Iberian margin and interpreted as a major decollement level or detachment fault at the top of serpentinized mantle^23–25^. Unit A discontinuity suggests that serpentinized mantle is exhumed in this area but only at one or two small windows (Fig. 2a) and that the observed tilted blocks represent allochthons of continental crust. Along Line 1, exhumed mantle and blocks of continental crust are sealed by post-tectonic sediments (unit B). However, going oceanward, the last allochthon block is over-tilted and the sedimentary unit B is here syn-tectonic (Fig. 2a,c), highlighting an oceanward migration of the deformation. This allochthon block is bounded by a major fault, which flattens and leads to mantle exhumation (Point β on Fig. 2a,b). We interpret it as a second major detachment system, cross-cutting the previous one. This interpretation is based on the fact that the pre-tectonic sediments (Unit A) overlying the tilted blocks of continental crust are not any more present oceanward above the basement. This observation suggests that the juxtaposed basement is younger than the deposition of the sedimentary unit A. Moreover, we interpret the over-tilted architecture of the latest continental block (Fig. 2c) as resulting from a new detachment fault cutting the first detachment fault and leading to mantle exhumation. Such a particular sedimentary architecture allowing the recognition of multiple detachment faults has been extensively detailed at the Australia-Antarctica margins by ref.^7^. A seamount is present in this hyper-extended continental domain on both seismic sections. The bright reflectivity and the passive infill against the flanks suggest that it is volcanic. These two edifices are clearly post-tectonic and post-unit B. However it is not clear if they are linked to the same event and to the margin evolution. Based on these observations it appears that the evolution of this domain is mainly controlled by tectonic processes, and particularly by multiple detachment faulting.

At the westernmost part of the profiles, the top basement morphology becomes smoother with high-amplitude reflections. The base of the crust is clearly marked by a set of reflections interpreted as oceanic Moho (Fig. 2d). These two parallel reflections define a 2 sec (TWT) thick basement, interpreted as the steady state oceanic crust.

## Architecture of the transitional domain

In between these two well defined hyper-extended continental and oceanic domains lies the transitional domain. The top basement is marked on both seismic sections by the presence of a volcanic edifice (V) (Fig. 2a,b). As the two lines cross-cut in this area, we suggest that we image here the same edifice. Volcanic lavas cover a top basement that is exhumed along detachment 2. Therefore the lavas are here clearly post detachment 2 and mantle exhumation. Immediately outboard of this volcanic edifice, we note a change in the top-basement morphology. We observe a step morphology of top basement associated with a thick sequence of high-amplitude reflectors very similar to SDRs (Seaward Dipping Reflectors) but which are here dipping toward the continent (Fig. 2e,f). These “Continentward Dipping Reflectors” (CDRs) downlap onto lavas from the adjacent volcanic edifice and onto a major, relatively flat, reflective interface. This reflector bends and deepens below the CDR sequence. Above this reflector lies an isolated faulted and tilted block (Fig. 2e,f) which seems to have been detached along this interface. This interface is thus interpreted as a third detachment fault (detachment 3) truncating detachment 2 and exhuming mantle rocks too. This tectonic event therefore precedes the emplacement of the volcanic edifice V and the emplacement of the CDRs sequence. The observed block above this detachment fault can be a rider block of serpentinized basement previously exhumed along detachment 2. It is completely buried under the CDRs sequence. The presence of these CDRs suggests an increase of the extrusive magmatic supply during the development of this transitional domain. Moreover, we observe the appearance of deep, discontinuous reflections at 10 sec TWT (Fig. 2e). They suggest the presence of underplated or intrusive magmatic material at depth. It is likely that the emplacement of these magmatic intrusions is related to the extrusive magmatic event leading to the CDRs sequence emplacement. This magmatic event seems thus to occur late, most likely after the development of detachment 3 and the formation of edifice V. The CDR sequence is finally affected by a normal fault (Fig. 2e,f), probably linked to late movement on the detachment fault. Except for this normal fault, no more deformation occurred at this time further inboard, highlighting the migration of the deformation toward the future ocean.

## Tectonic model and processes controlling lithospheric breakup

**Figure 3:**
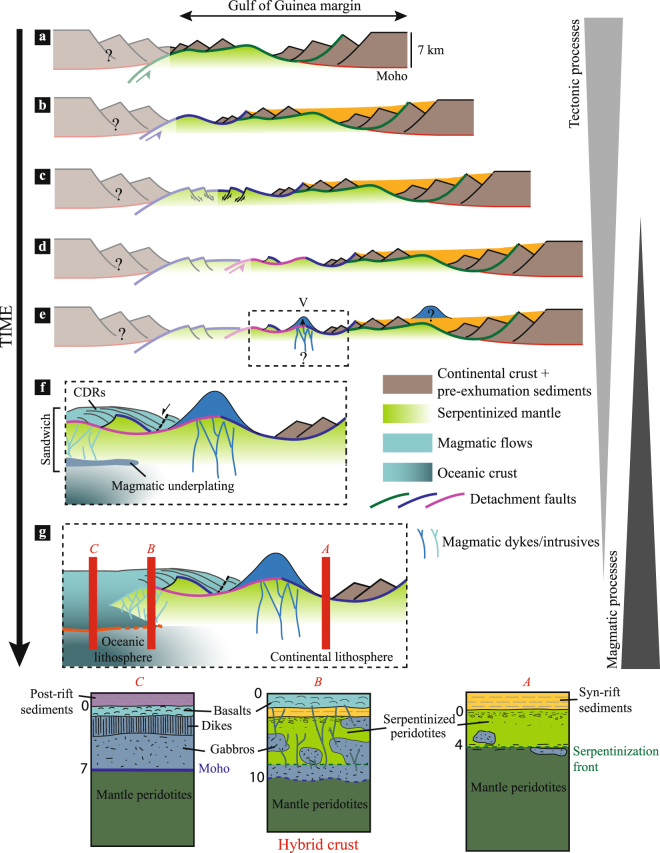
Conceptual, evolutionary model highlighting the transition from tectonic to magmatic-driven processes. Transparent left part represents the conjugate margin. (**a**) Development of the first detachment system (green). (**b**) Development of the second detachment system (blue) leading to mantle exhumation. (**c**) Normal faulting of the previously exhumed basement. (**d**) Development of the third detachment system (pink). (**e**) Emplacement of the volcanic seamount V above previously exhumed basements. (**f**) Focus on the evolution of the transitional domain with the emplacement of the CDRs at the top basement and magmatic underplating (sandwich architecture). (**g**) Lithospheric breakup is achieved, emplacement of the steady state oceanic crust. Three logs summarizing the different basement architectures and highlighting their potential real complexity.

High resolution seismic imaging shows that the transition into first undisputable oceanic crust is marked by “existing” hyperextended curst or exhumed mantle sandwiched in between new magmatic additions. This architecture highlights the idea of a “hybrid” transitional crust^7,15,26^. Thus, the creation of first oceanic crust results from a competition between dying tectonic and nascent magmatic processes. Tectonic processes and in particular detachment faulting drive the separation of continental crust and the creation of new basement, i.e. creation of exhumed mantle (Fig. 3). Similar processes are observed at ultra-slow spreading ridges leading to the creation of an exhumed oceanic basement^27–29^. However, in contrast to steady state oceanic domains where deformation and magmatic systems remain localized within the spreading axis, the deformation in rift systems is not yet localized as indicated by the in- and out-of-sequence stepping of multiple detachment faults^8^.

The evolution of magma-poor margins is characterized by the thinning of the continental lithosphere that is associated with multiple, polyphase tectonic events, with no or little magmatic supply (Fig. 3a–c). However, the rise of the asthenosphere progressively leads to the production of magma that infiltrates in the overlying continental lithospheric mantle^30^, resulting in a combined tectonic and thermal thinning of the lithosphere. This critical stage marks the onset of the lithospheric breakup (Fig. 3d–f). While at magma-rich systems magma production and extraction may be sufficient to directly rupture the lithosphere, at magma-poor systems tectonic processes (e.g. detachments faults) continue to accommodate extension in the presence of a stuttering magmatic system^10,31^ (Fig. 3d,e). The volcanic edifice V may result from a magmatic pulse during this stage (Fig. 3e). The increase of the magmatic supply, indicated by the emplacement of CDRs above previously exhumed basement (Fig. 3f) shows, however, that magmatic processes become progressively more dominant. Although this volcano-sedimentary sequence appears mostly post-tectonic relative to detachment fault 3, the late normal fault affecting the CDRs indicates that tectonic movements continue even after onset of more dominant magmatic activity. This polyphase tectono-magmatic activity shows that the deformation is not yet localized at a spreading center and that the lithospheric breakup is not yet achieved. Even though the interpretation of magmatic intrusive rocks is difficult in seismic sections, the large amount of extracted magma makes it likely that the observed deep reflections in the transitional domain may correspond to the stagnation of magmatic rocks at depth. In the case of initial mantle exhumation, later magmatic additions may result in “underplated” bodies at the neutral buoyancy level (density of magma equals density of host rock) or at a hydrothermal boundary (serpentinization front) (Fig. 3f). The “sandwich” architecture thus results from the stacking of previously exhumed mantle and new extrusive and underplated magmatic material (Fig. 3f). This overlapping of processes results in the formation of a “hybrid” crust. It highlights the fact that the lithospheric breakup is not a brutal event at magma-poor rifted margins, neither in time nor in space, but rather the result of a gradual transition from tectonic to magma-dominated processes^8,15^ (Fig. 3g). The transitional domain could thus represent the spatial extension of the lithospheric breakup. It leads to the creation of a new plate boundary, which appears to be diffuse rather than sharp. The final architecture and the width of the transition will depend on the capacity to create and efficiently extract magmatic material during lithospheric thinning. Differences of architecture between magma-poor and magma-rich margins could be linked to these variations.

Our observations show that the emplacement of the first oceanic crust at magma-poor rifted margins results from a polyphase and gradual evolution, with tectonic processes “passing on the baton” to the nascent magmatic system. The resulting transitional hybrid domain certainly marks the occurrence of the breakup of the continental lithosphere and the creation of a new, diffuse, plate boundary. These results bring new constraints on lithospheric breakup processes and on the architecture of the associated domain for magma-poor rifted margins. It is of particular interest for paleogeographic reconstructions as it highlights the need to be careful in reconstructing transitional and inboard domains.

### Data availability

The data that support the findings of this study are available from Petroleum Geo-Services (PGS) but restrictions apply to the availability of these data, which were used under license for the current study, and so are not publicly available.

## Electronic supplementary material

Seismic line drawing
