# Supplementary material for: Birth of an oceanic spreading center at a magma-poor rift system
Source: Sci Rep. 2017 Nov 8;7:15072. doi: 10.1038/s41598-017-15522-2 (PMC5678130; doi:10.1038/s41598-017-15522-2)
Supplement: Supplementary file 1 — Seismic line drawing [file 41598_2017_15522_MOESM1_ESM.pdf]

Supplementary Information

Birth of an oceanic spreading center at a magma-poor rift system

Morgane Gillard[1]*, Daniel Sauter[1], Julie Tugend[1], Simon Tomasi[1], Marie-Eva Epin[1], Gianreto Manatschal[1]

[1]Institut de Physique du Globe de Strasbourg, UMR7516, Université de Strasbourg/EOST, CNRS, 1 rue Blessig, Strasbourg Cedex F-67084, France
*Correspondence to mgillard@unistra.fr

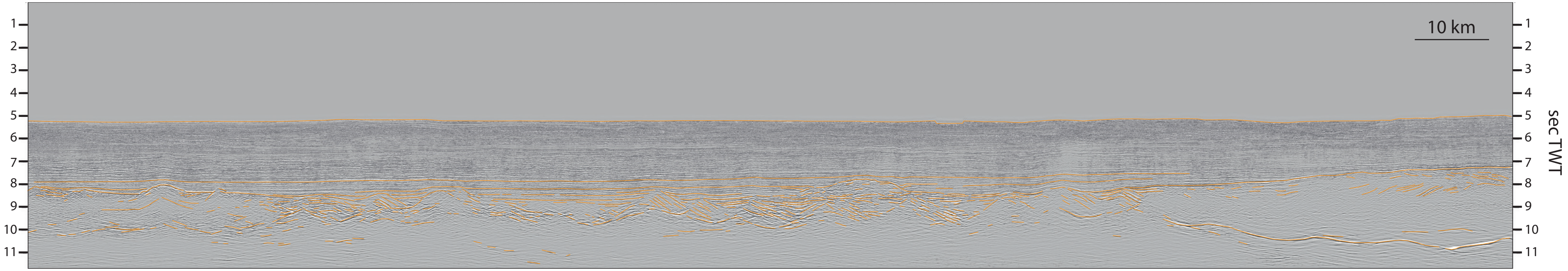

Line 1

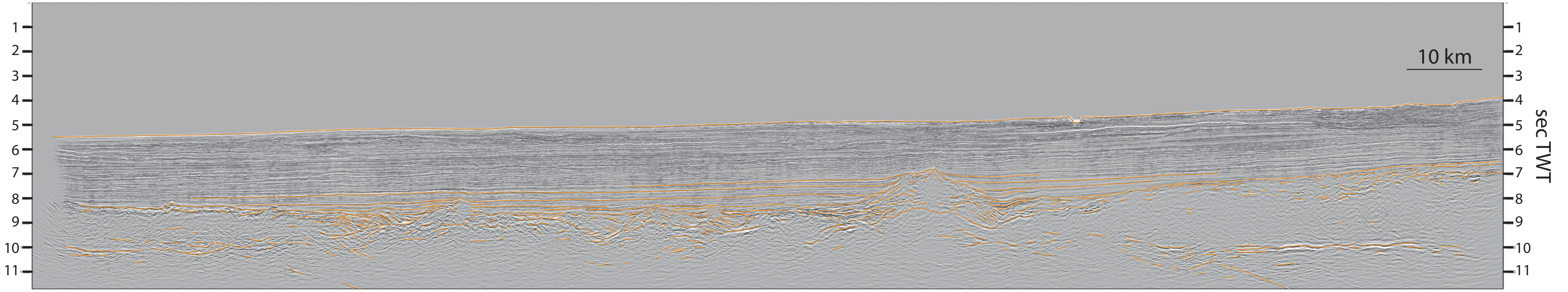

Line 2
